# Identification of therapeutic sensitivities in a spheroid drug combination screen of Neurofibromatosis Type I associated High Grade Gliomas

**DOI:** 10.1371/journal.pone.0277305

**Published:** 2023-02-02

**Authors:** Jacquelyn Dougherty, Kyra Harvey, Angela Liou, Katherine Labella, Deborah Moran, Stephanie Brosius, Thomas De Raedt

**Affiliations:** 1 Department of Pediatrics, Children’s Hospital Philadelphia, Philadelphia, Pennsylvania, United States of America; 2 School of Medicine, University of Pennsylvania, Philadelphia, Pennsylvania, United States of America; 3 Department or Neurology, Children’s Hospital Philadelphia, Philadelphia, Pennsylvania, United States of America; Goethe University Hospital Frankfurt, GERMANY

## Abstract

Neurofibromatosis Type 1 (NF1) patients develop an array of benign and malignant tumors, of which Malignant Peripheral Nerve Sheath Tumors (MPNST) and High Grade Gliomas (HGG) have a dismal prognosis. About 15–20% of individuals with NF1 develop brain tumors and one third of these occur outside of the optic pathway. These non-optic pathway gliomas are more likely to progress to malignancy, especially in adults. Despite their low frequency, high grade gliomas have a disproportional effect on the morbidity of NF1 patients. *In vitro* drug combination screens have not been performed on NF1-associated HGG, hindering our ability to develop informed clinical trials. Here we present the first *in vitro* drug combination screen (21 compounds alone or in combination with MEK or PI3K inhibitors) on the only human NF1 patient derived HGG cell line available and on three mouse glioma cell lines derived from the NF1-P53 genetically engineered mouse model, which sporadically develop HGG. These mouse glioma cell lines were never exposed to serum, grow as spheres and express markers that are consistent with an Oligodendrocyte Precursor Cell (OPC) lineage origin. Importantly, even though the true cell of origin for HGG remains elusive, they are thought to arise from the OPC lineage. We evaluated drug sensitivities of the three murine glioma cell lines in a 3D spheroid growth assay, which more accurately reflects drug sensitivities *in vivo*. Excitingly, we identified six compounds targeting HDACs, BRD4, CHEK1, BMI-1, CDK1/2/5/9, and the proteasome that potently induced cell death in our NF1-associated HGG. Moreover, several of these inhibitors work synergistically with either MEK or PI3K inhibitors. This study forms the basis for further pre-clinical evaluation of promising targets, with an eventual hope to translate these to the clinic.

## Introduction

Neurofibromatosis 1 (NF1) is an autosomal dominant tumor predisposition syndrome impacting approximately one in every 3000 births [1–3]. Clinical manifestations of NF1 include a broad array of benign and malignant tumors of the peripheral and central nervous system (CNS). CNS tumors include optic pathway glioma, non-optic pathway low-grade gliomas (LGG), and high-grade gliomas (HGG) [4, 5]. About 15–20% of NF1 patients develop glioma, the majority of which comprise of LGG (~17%) [6, 7]. NF1-associated HGG (NF1-HGG) develop in ~2% of patients and have significant mortality and morbidity. To date, NF1-associated HGG is incurable with a 5-year overall survival of 5%, despite advances in neurosurgical, radiation and chemotherapeutic approaches [7], highlighting the need to develop effective therapies for NF1 patients with HGG.

NF1 individuals have a germline mutation in the NF1 gene that encodes neurofibromin, a GTPase activating protein that negatively regulates the RAS pathway. Mechanistically, neurofibromin accelerates the conversion from the active GTP-bound RAS to its inactive GDP-bound form. NF1-HGG have complete functional loss of the *NF1* tumor suppressor gene due to inactivating mutations or deletions [8], leading to an aberrant over-activation of the RAS signaling cascade and increased downstream activity of the oncogenic PI3K-AKT and MEK-ERK effector pathways [9]. Recently, genetic profiling of NF1-HGG has identified frequent mutations in ATRX (an epigenetic modifier), CDKN2A and TP53 [7, 10].

To date, limited drug sensitivity studies have been performed on NF1 associated High Grade Gliomas. This is in part due to the lack of HGG cell lines derived from NF1 patients; only a single human NF1-HGG is publicly available (TM-31). This cell line has inactivating mutations of NF1 (pLF1247fs*18, homozygous), ATRX (E2281*, homozygous), CDKN2A (homozygous deletion) and TP53 (C238G) (https://cellmodelpassports.sanger.ac.uk/passports/SIDM01460). To our knowledge, this is the first drug sensitivity study performed on a HGG cell line derived from an NF1 patient.

Genetically engineered mouse models for NF1 are available. Intriguingly, about 10% of NPcis mice (*Nf1*+/- and *Tp53*+/- in cis [11]) sporadically develop HGG that can be used as a proxy for human lines. We have generated cell lines from HGGs that developed in three NPcis mice (17, 5653 and 5746). Crucially, our mouse lines have never been exposed to serum, are grown in neuronal stem cell media and express markers consistent with an OPC identity. Non-serum grown glioma lines better recapitulate the tumor biology than glioma cell lines exposed to serum [12]. Signaling pathways and drug sensitivities can differ between cells grown in a monolayer or as spheres, with the belief that 3D growth more accurately reflects human disease [13–15]. Our mouse glioma cell lines naturally grow as spheres; hence, our mouse drug sensitivity studies were performed in 3D growth assays. As the human NF1-HGG TM-31 cell line is an adherent line grown in media containing serum, drug sensitivities were evaluated in a monolayer (2D) culture.

In our study, we sought to identify NF1-HGG sensitivities against agents targeting downstream RAS effector pathways (MAPK and AKT pathways) in combination with a broad array of approved and investigational anti-cancer drugs. Although NF1-HGG arises from aberrant RAS pathway hyperactivation, it is well established that monotherapy targeting a single pathway often renders limited therapeutic benefit especially against malignant, high-grade tumors. In recent years, combinatorial drug therapies have significantly altered the prognosis of many cancers in children and adults [16]. Considering this, sensitivity to 21 cancer drugs, that are in clinical development, was evaluated alone and in combination with inhibition of the RAS-MAPK or RAS-PI3K-AKT pathways. Specifically, drug sensitivities against monotherapy PI3K (GDC0941) and MEK (Trametinib) inhibition were determined and followed by combinatorial drug treatments with a panel of anti-neoplastic agents. Combining inhibition of downstream RAS effector pathways with Vorinostat (HDAC inhibitor), LY2606368 (CHEK1 inhibitor), PTC596 (BMI-1 inhibitor), JQ1 (BRD4 inhibitor), Dinaciclib (CDK1/2/5/9 inhibitor) and Bortezomib (proteasome inhibitor) caused potent cell death. These pairs form novel and powerful drug combinations that will require further preclinical investigation to drive future translation into effective therapeutic strategies for NF1-HGG patients.

## Material and methods

### Ethics statement

This research was determined to be none-human subject research by the Children’s Hospital Philadelphia Committees for the Protection of Human Subjects (IRB). The need for consent was waived.

### Cell lines and materials

The NF1 patient-derived HGG cell line TM-31 (RRID:CVCL_6735) [17], purchased from the RIKEN biorepository, is null for NF1, TP53, CDKN2A and ATRX. The Glioblastoma Multiphorme (GBM) LN319 cell line was a gift from Dr. Karen Cichowski. LN319 is an adult sporadic GBM with full inactivation of NF1. TM-31 and LN319 cells are grown in Dulbecco’s Modified Eagle’s Medium (DMEM; Gibco, Life Technologies, NY, USA) supplemented with 10% fetal bovine serum (FBS; Gemini-Bio Products, West Sacramento, CA, USA), 1% glutamax (Gibco) and 1% Penicillin/Streptomycin (Life Technologies, NY, USA).

Mouse NF1-HGG cell lines 17, 5653, and 5746 were derived from three distinct high-grade murine brain tumors sporadically developed by the NPcis genetically engineered mouse model. The germline of these mice is heterozygous for *Nf1* and *Tp53*; mutant alleles are located in cis. The 17, 5653, and 5746 glioma cell lines are grown in NeuroCult^™^ Mouse and Rat Basal Medium (NeuroCult; StemCell Technologies, Vancouver, BC, Canada) supplemented with NeuroCult^™^ Mouse and Rat Proliferation Supplement (StemCell Technologies, Vancouver, BC, Canada), 1% Penicillin Streptomycin (P/S; Life Technologies, NY, USA), 20 ng/mL human recombinant epidermal growth factor (EGF; StemCell Technologies, Vancouver, BC, Canada) and 10 ng/mL human recombinant fibroblast growth factor (FGF; StemCell Technologies, Vancouver, BC, Canada).

All cells are cultured at 37°C in a humidified 5% CO2 incubator. Cell lines were transduced with Incucyte^®^ Nuclight Red Lentivirus (Sartorius, Gottingen, Germany) such that they will produce an EF1α promoter driven nuclear restricted red fluorescent label (nuclear RFP) and flow sorted to select for successfully transduced cells (Sartorius, Gottingen, Germany).

The following antibodies were purchased from Cell signaling Technologies: pERK (Catalogue # 4370), total ERK (Catalogue # 9102), pAKT (Catalogue # 4060), total AKT (Catalogue # 4691), Ubiquitin (Catalogue # 4289), MYC (Catalogue # 18583), pCHEK1 S296 (Catalogue # 90178), BMI1 (Catalogue # 5856), H3K27Ac (Catalogue # 8173), total H3 (Catalogue # 14269), CDK9 (Catalogue # 2316), Vinculin (Catalogue # 4650), CPNase (Catalogue # 5664) and MAG (Catalogue # 9043). Beta-Actin (Catalogue # NB600-501) was purchased from Novus, pS2 RNApol II (Catalogue # 04-1571-1) from EMD Millipore, SOX2 (Catalogue # MAB2018) from R&D systems and NG2 (Catalogue # PA5-17199) from Invitrogen.

### Compound used in screen

All drugs and compounds were purchased from Selleckchem (Houston, TX, USA). A complete list of compound names, sources, catalogue numbers, are listed in S1 Table. These compounds encompass a broad spectrum of molecular targets that have been implicated in cancer and are either in preclinical studies or in clinical development.

### Spheroid drug sensitivity assay

Two thousand cells/well of 17, 5653, or 5746 glioma cell lines are plated in 96-well round-bottomed ultra-low attachment plates (MS9096UZ, S-bio, Hudson, NH) and used for spheroid drug screening. Cells are plated in 100uL of complete culture media, centrifuged (90g, 5min) and incubated overnight to stimulate a single colony from forming at the bottom of the round well. Twenty-one drugs were screened, using three concentrations (see S2 Table, in triplicate) and prepared in NeuroCult media. All drugs were combined with DMSO, a single concentration of Trametinib (10nM), or a single concentration GDC0941 (1000nM for 17 and 5746, 500nM for 5653). For each cell line, these concentrations of Trametinib and GDC-0941 were chosen to be below the Half Maximal Inhibitory Concentration (IC50) value, in order to discern the maximum cooperativity between drug combinations. The final volume of media for each well is 150ul. Each concentration of a drug combination was evaluated in triplicate, including the DMSO controls. The total integrated intensity of RFP was monitored over the course of 72 hours, taking measurements every 2 hours with the Incucyte^®^ S3 Live-Cell Analysis System (Sartorius, Gottingen, Germany) and serve as a proxy for the number of cells present. Analysis parameters are listed in S3 Table.

### 2D drug sensitivity assays

The TM-31 cells were plated (10,000 cells/well) in 96-well flat-bottom tissue culture treated plates (Corning 3596). Cells were plated in 100uL culture media and incubated overnight before adding drugs on Day 0. Twenty-one drugs were screened, using three concentrations (see S2 Table, in triplicate) and prepared in DMEM media (10% FBS). All drugs were combined with DMSO, a single concentration of Trametinib (20nM), or a single concentration of GDC0941 (500nM). These concentrations of Trametinib and GDC-0941 were chosen to be below the IC50 value, in order to discern the maximum cooperativity between drug combinations. The total volume of media per well is 150ul. Each concentration of a drug combination was evaluated in triplicate, including the DMSO controls. We monitored cell numbers over the course of 72 hours, taking measurements every 2 hours with the Incucyte^®^ S3 Live-Cell Analysis System (Sartorius, Gottingen, Germany). We used the Incucyte^®^ S3 Live-Cell Analysis software to identify and count the number of individual cells, as defined by the number of RFP^+^ nuclei, across the 72 hours. Analysis parameters are listed in S4 Table.

### Data analysis

The log2-fold change in total integrated intensity or cell number (for 3D and 2D assays, respectively) for each time point relative to 0 hours was calculated. Drugs were considered for a broader follow-up combinatorial evaluation if our initial treatment caused more than 50% cell death in 72h (Log2 fold change value <-1) at concentrations below the maximum serum concentration that can be safely attained during pharmacokinetic drug studies for clinical trials (Cmax). These Cmax obtained from the literature (Table 2), and values were converted from mg/ml to nM.

### Synergy screening

The synergy of drug combinations was evaluated in the mouse 5746 and TM31 glioma cell lines. The 17 glioma cell line was used for the PTC596/Trametinib combination as this combination showed limited sensitivity than the 5746 line (S2 Table). Cell lines were plated in 96-well plates and processed as described in the spheroid or 2D drug screening assays. We created a dilution series of our drug combinations (7 dilutions of the drug of interest, 5 dilutions of Trametinib or GDC-0941). On Day 0, Trametinib, GDC-0941, Bortezomib, Dinaciclib, JQ1, LY2606368, PTC596, or Vorinostat were added in a serial dilution per row in duplicate. We used the percent viability compared to untreated cells to calculate a synergy score for each of the analyzed combinations (https://synergyfinder.fimm.fi/; parameters: LL4 curve fit, ZIP method for synergy score calculation).

### Statistical analysis

Our initial 21 compound drug screen was performed in triplicates, the synergy study was performed in duplicates. IC50 and Lethal Dose 50 (LD50) values and 95% confidence intervals were calculated to assess drug sensitivity (GraphPad Prism 9 software) and tabulated in Table 1.

**Table 1:** Synergistic evaluations of drug combinations in 5746 and TM31 cell lines.

5746		Single compound				Trametinib (10nM)			GCD0941 (1uM)		
Drug	Target	IC50 (nM)	CI	LD50 (nM)	CI	LD50 (nM)	CI	Synergy	LD50 (nM)	CI	Synergy
Trametinib	MEK	17	15–20	>40	NC	-	-	-	9.6	8.8–10.6	Syn (26.1)
Vorinostat	HDAC	535	451–632	1217	1113–1341	733	671–804	Add (4.2)	443	415–474	Syn (12.6)
JQ1	BRD4	415	385–446	>2000	NC	740	637–866	Syn (12.7)	424	399–451	Add (9.7)
LY2606368	CHK1	35	29–45	>100	NC	>100	NC	Syn (18.0)	10.2	8.9–11.8	Syn (20.6)
PTC596	BMI1	66	57–78	>500	NC	168*	153–184*	Syn (13.9)*	129	97–172	Syn (18.8)
Bortezomib	proteasome	4.3	4.1–4.5	5.6	4.9–6.3	5.1	4.5–5.7	Add (0.4)	4.1	3.5–4.8	Add (1.9)
Dinacicilb	CDK1/2/5/9	26.4	10–30.1	25	13–40	26	22.6–32.0	Add (-3.9)	19.6	19–20	Add (6.1)
TM31NR		Single compound				Trametinib (25nM)			GCD0941 (1uM)		
Drug	Target	IC50 (nM)	CI	LD50 (nM)	CI	LD50 (nM)	CI	Synergy	LD50 (nM)	CI	Synergy
Trametinib	MEK	78	51–139	>200	NC	-	-	-	>200	NC	Add(8.5)
Vorinostat	HDAC	646	515–806	1973	1782–2201	1340	1087–1645	Add (1.8)	1410	969–2017	Add(2.4)
JQ1	BRD4	580	418–819	>4000	NC	>4000	NC	Add (-5)	>4000	NC	Add(-0.1)
LY2606368	CHK1	1.9	0.4–6.2	136	89–241	>1000	NC	Ant(-12.5)	272.5	153->1000	Add(-5.1)
PTC596	BMI1	38	28–50	107	85–140	95	84–108	Add(1.5)	90	81–102	Add(-1.7)
Bortezomib	proteasome	6.4	5.6–7.4	10.4	9.7–11.1	11.8	10.6–13.1	Add(-4)	10.2	9.7–10.6	Add(-2.5)
Dinacicilb	CDK1/2/5/9	9.3	5.8–14.1	25	22–30	21.7	21–22	Add(-0.8)	16.8	16–17	Add(1.4)

NA: not available; IC50: 50% growth inhibition; LD50: Lethal dose 50 (50% cell death), NC: Not Calculated, Synergy: Synergy score listed between brackets, Syn: synergistic, Add: additive, Ant: antagonistic CI: Confidence interval;

* Determined in mouse 17 cell line

### Cell line characterization and drug target inhibition studies

To characterize our mouse glioma cell lines, a genotyping PCR for *Nf1* (primers: Nf1-wt-F: GGTATTGAATTGAAGCAC, Nf1-R: TTCAATACCTGCCCAAGG, Nf1-mut-F: ATTCGCCAATGACAAGAC) and *Tp53* (primers: Tp53-wt-F: AGGCTTAGAGGTGCAAGCTG, Tp53-R: TGGATGGTGGTATACTCAGAGC, Tp53-mut-F: CAGCCTCTGTTCCACATACACT) was performed [11]. The reported ATRX mutation (E2281*, PCR primers: GAACATGATTCTCTTTTGGACCAC and ACCTGTTTCAAATGTGACCCTTT and sequencing primer GGATACCATACTTGCAGAGC) and NF1 mutation (p.LF1247fs18*, primers: AATAAAAATGGGATTGTTTG and GGAAGAGAGTCTGCATGGAG) in the TM-31 cell line was confirmed by sequencing. Western blot was performed to evaluate the expression of OPC lineage markers and for the expression of TP53 and CDKN2A in TM-31. Target inhibition of the drugs that caused potent cell death in our glioma lines inhibited was evaluated by western blot as well. Glioma cell line 17 was treated for 24h with 20nM Trametinib, 1μM GDC-0941, 5nM bortezomib, 25nM Dinaciclib, 500nM JQ1, 100nM LY2606368, 500nM PTC596, or 1μM Vorinostat and target inhibition was evaluated by comparing the expression levels of pERK, pAKT, Ubiquitin, pS2 RNApol II and CDK9, MYC, pCHEK1, BMI1 and H3K27Ac respectively.

## Results

### Cell line validation and characterization

**Fig 1:**
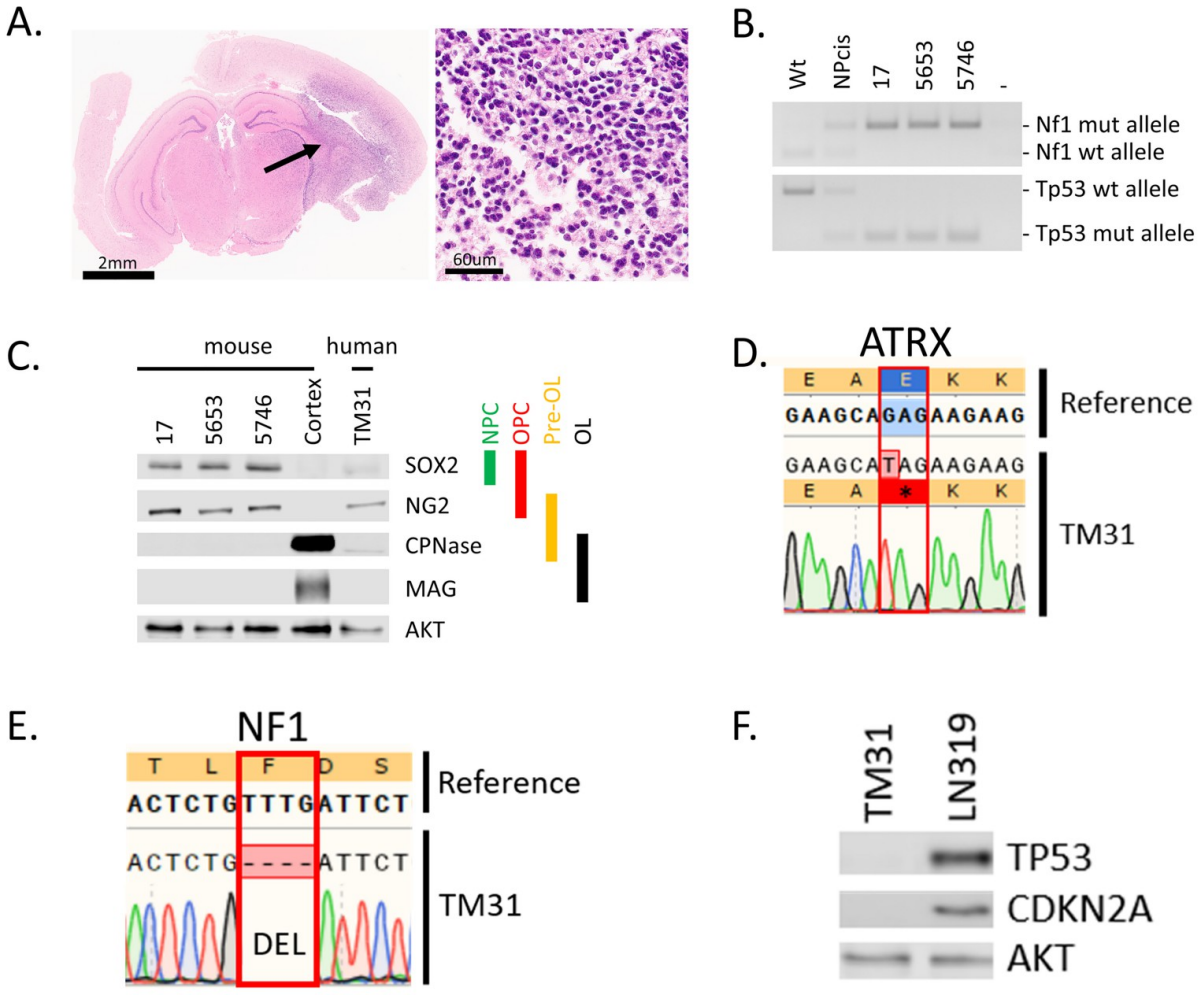
Characterization of cell lines. (A) H&E staining of tumor 5653, the arrow indicates the location of the tumor. The right panel shows a higher magnification. (B) Genotyping PCR on genomic wildtype mouse, genomic NPcis mouse (heterozygous for *Nf1* and *Tp53*) DNA and DNA extracted from cell lines 17, 5653 and 5746. The glioma cell lines show complete loss of the wildtype allele for Nf1 and Tp53. (C) Western blot shows the expression of markers consistent with OPC lineage origin of our cell lines. SOX2 is expressed in Neural Progenitor cells (NPC) and Oligodendrocyte precursor cells (OPC); NG2 is expressed in OPCs. Markers (CPNase and MAG) expressed in pre-oligodendrocytes (pre-OL) or mature oligodendrocytes (OL) are not present, highlighting the progenitor status of our lines [22]. (D) The ATRX mutation (E2281*) present in TM31 was confirmed using sequencing. Top shows the reference genome DNA and protein sequence, bottom shows the TM31 sequence with STOP mutation. (E) The homozygous 4bp NF1 deletion (p.LF1247fs*18) present in TM31 was confirmed using sequencing. Top shows the reference genome DNA and protein sequence, bottom shows the TM31 sequence with the homozygous 4bp deletion inducing a frameshift. (F) Western blot for TP53 and CDKN2A, showing that both genes are absent in TM31, but not in glioma line LN319.

Our mouse NF1-HGG cell lines were derived from 3 gliomas that sporadically arose in our NPcis mouse model [11, 18]. These mice have a germline mutation in both *Nf1* and *Tp53*, in cis on mouse chromosome 11. Tumors form when a cell loses the wildtype copy of Nf1 and *TP53*, usually due to whole chromosome loss. Importantly, the loss of the wildtype chromosome occurs sporadically, each tumor thus is unique. About 10% of these NPcis mice form high grade gliomas [18]. Fig 1A shows the H&E staining of tumor 5653 with HGG morphology. We did not observe necrosis in these tumors, suggesting these are HGG and not GBM. All mouse glioma lines show full loss of *Nf1* and *TP53* as evidenced by the loss of the wildtype *Nf1* and *Tp53* alleles of the genotyping PCRs (Fig 1B). Finally, these glioma cell lines express the oligodendrocyte precursor cell (OPC) markers SOX2 and NG2 (Fig 1C), consistent with the suspected cell of origin for HGG [19–21].

Our human NF1-HGG line was derived from a malignant astrocytoma of a 42-year-old female Neurofibromatosis Type I patient and has full loss/inactivation of *NF1*, *ATRX*, TP53 and *CDKN2A* (Fig 1D–1F). These genes are frequently lost in NF1-associated HGG. We confirmed the identity of this cell line by sequencing the homozygous ATRX STOP mutation (E2281*) (Fig 1D) and the homozygous NF1 4bp deletion causing p.LF1247fs*18 (Fig 1E) and showed loss of CDKN2A and TP53 by western blot (Fig 1F). Importantly, even though this line is grown in serum conditions, which induces differentiation, markers consistent with OPC identity are still expressed (Fig 1C).

### Sensitivity to MEK and PI3K inhibitors

**Fig 2:**
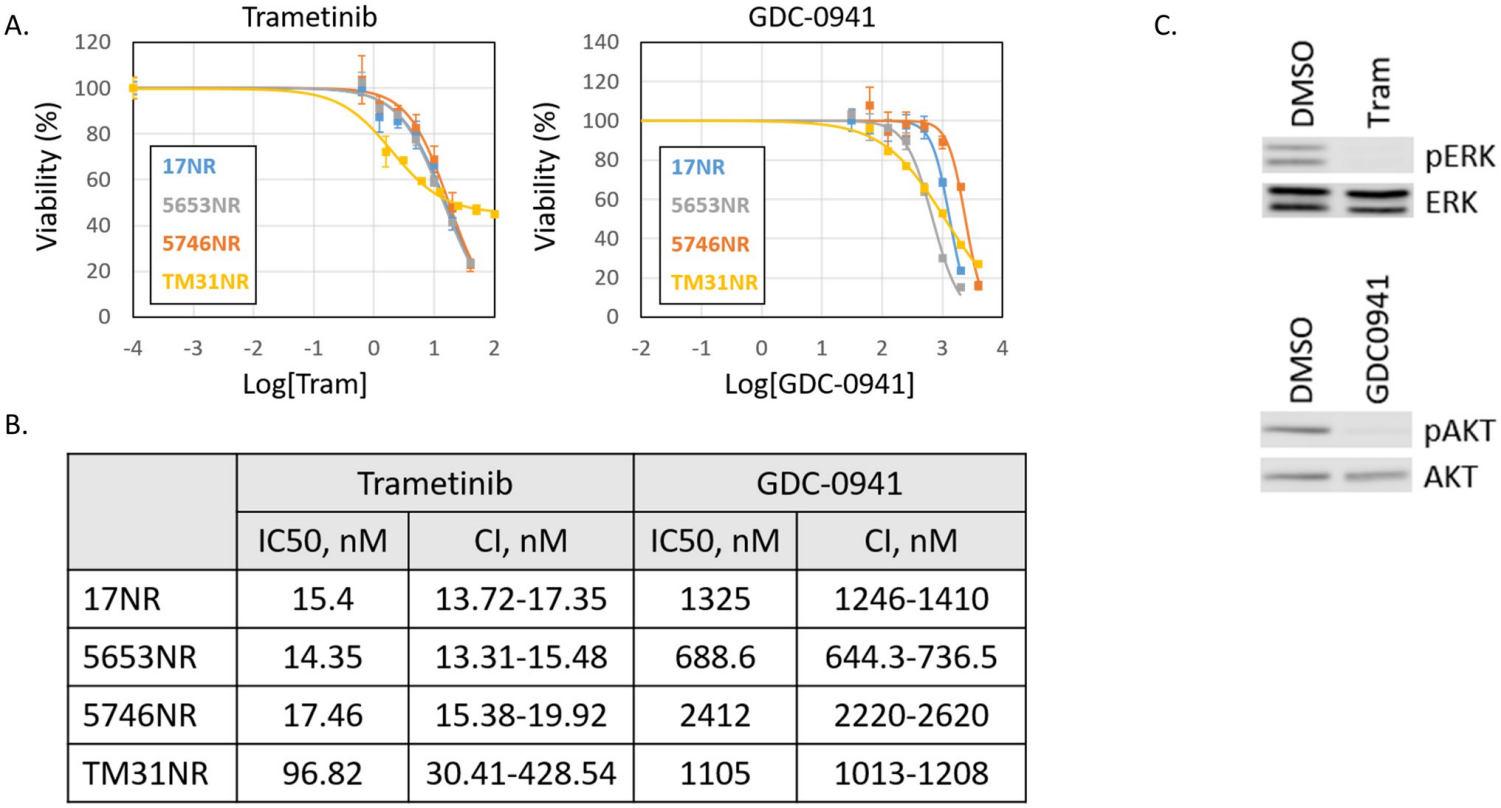
Sensitivity to MEK and PI3K inhibitors. (A) Dose response curves to the MEK inhibitor Trametinib and PI3K inhibitor GDC-0941 of our NF1 associated HGG cell lines. Error bars denote the standard deviation. NR denotes expression of Nuclear Red fluorescence marker (B) IC50 values and 95% confidence interval (CI) for Trametinib and GDC-0941. (C) Western blot of pERK inhibition by Trametinib (20nM) and pAKT inhibition by GDC-0941 (1μM) in our 17 cell line.

Given that our HGG cell lines are driven by NF1 loss and the consequent hyperactivation of RAS, agents that antagonized the two main RAS effector pathways, the PI3K inhibitor Pictilisib (GDC-0941) and the MEK inhibitor Trametinib (GSK1120212) were selected. Both agents are currently in active or recently completed clinical trials for solid and brain malignancies. Fig 2A shows the dose response curves of our NF1 glioma lines to single agent MEK or PI3K inhibition (72h treatment). IC50s varied between 688 and 2412nM for GDC-0941. The IC50s for Trametinib varied between 14 and 17nM for the mouse glioma cell lines and is 97nM for the human serum grown TM-31 line (Fig 2B). Fig 2C shows that at the concentrations used we have near full inhibition of pERK (as a readout of MEK inhibition) and pAKT (as a readout for PI3K inhibition).

### MEK and PI3K inhibitor combination drug screen

**Fig 3:**
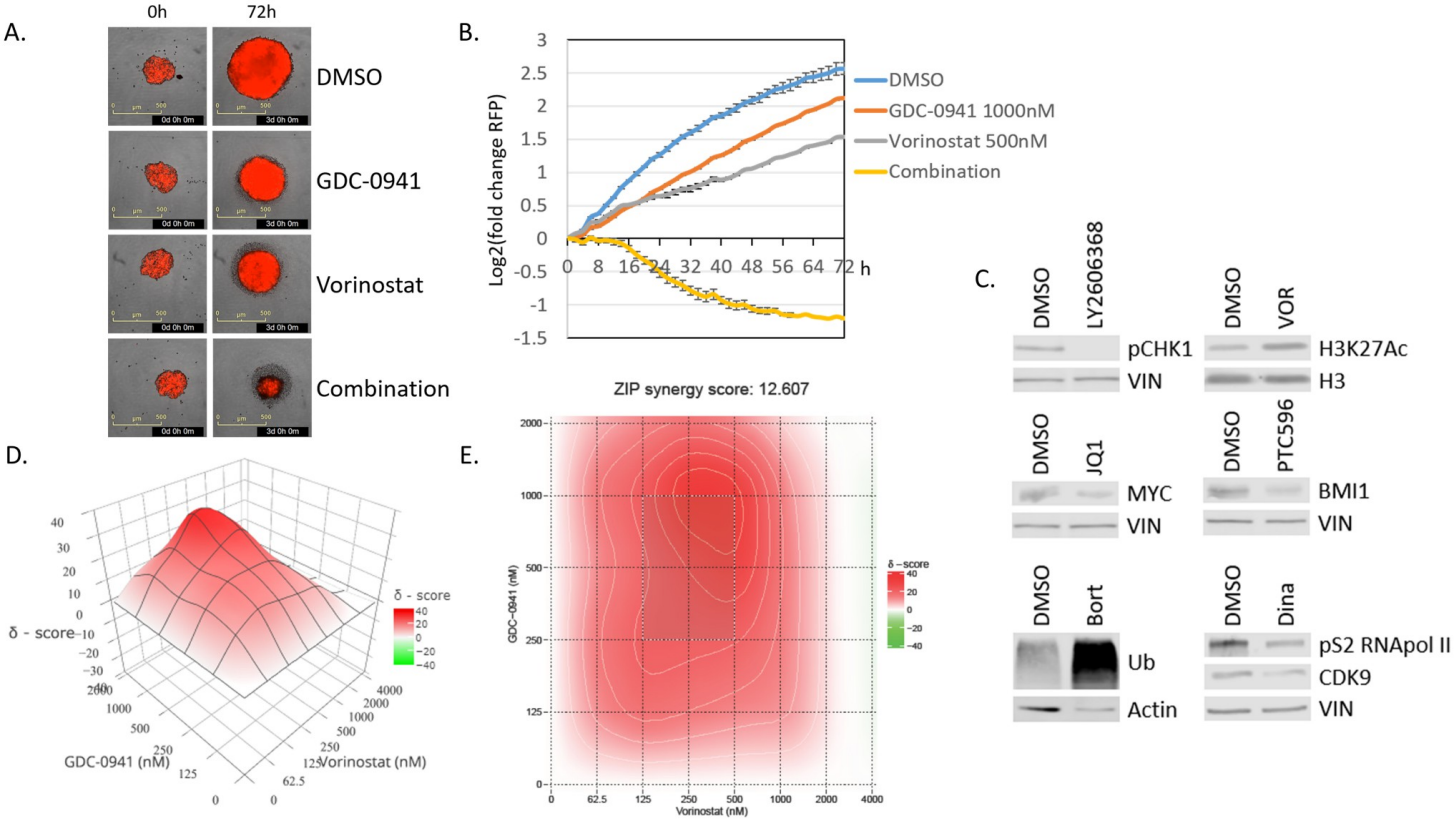
Sensitivity to combination therapies. (A) Representative images of glioma line 5746 treated with GDC-0941 (1000nM), Vorinostat (500nM) or a combination. Images on the left show size and red intensity of the sphere at the starting point of the assay (0h). Images on the right show the same sphere at 72h. Combination of GDC-0941 and Vorinostat causes potent cell death. (B) Growth curves of 5746 treated with GDC-0941 and/or Vorinostat for 72h. Error bars denote the standard deviation. A log2(fold change) of -1 denotes 50% lower signal compared to starting point. (C) Western blot confirming the target inhibition of our compounds. An increase in ubiquitinated proteins is shown by treatment of cell line 17 with 5nM bortezomib; pCHEK1 is inhibited with 100nM LY2606368; MYC [33] is inhibited by the BRD4 inhibitor JQ1 (500nM); BMI1 [34] is downregulated by treatment with 500nM PTC596; H3K27Ac is upregulated by treatment with 1μM Vorinostat and expression of CDK9 [35] and its substrate pS2 RNApol II is reduced with 25nM Dinaciclib. (D) 3D graphical representation of the synergy score as determined by synergy finder. X-axis: GDC-0941 concentration, Y-axis Vorinostat concentration, Z-axis synergy score (δ). (E) 2D heatmap representing the same synergy score data (D).

The sensitivity to 21 compounds as single agents and in combination with Trametinib or GDC-0941 was determined. S2 Table shows the log2 fold change data in RFP signal (spheroid assays) or number of cells (2D assays). Positive values (green) denote cell growth; negative values (red) denote cell death, with fewer cells present compared to 0h. Thirteen drug combinations passed our criteria as a combination of interest (more than 50% cell death i.e. a Log2 fold change <-1 at a concentration lower than the Cmax, the maximum safe blood concentration reached in clinical pharmacokinetic experiments) and will be evaluated to determine if these effects were additive or synergistic. Table 1 shows the IC50 and LD50 values in the 5746 or 17 cell line of the 6 compounds (Vorinostat, Bortezomib, JQ1, LY2606368, PTC596 and Dinaciclib), that induced potent cell killing across our cell lines at concentrations below the Cmax. Importantly, the IC50 value denotes the concentration where we observe 50% reduced growth compared to controls, where the LD50 denotes 50% fewer cells compared to the start of the assay and is a measure for induced cell death. The LD50 of the combination of Trametinib with GDC-0941 is also shown. Importantly, for all of these agents, our cells had LD50 values (i.e. 50% cell death) at or below the IC50 values (i.e. 50% growth inhibition) of the 25% most sensitive cancer cell lines as reported by The Genomics of Drug Sensitivity in Cancer Project (https://www.cancerrxgene.org/compounds) or in the literature [23, 24] (Table 2), highlighting the sensitivity of our lines. Fig 3A and 3B show an example of the potent cell killing induced by a combined treatment of GDC-0941 (1000nM) and Vorinostat (500nM) of the 5746 line. Target inhibition of all compounds was confirmed by western blot, Fig 3C.

**Table 2:** Cmax and 25^th^ percentile IC50 values across cancer cell lines.

Drug	Target	Cmax (nM)	25th percentile of IC50s across cancer cell lines^+^
Trametinib	MEK	36 [25]	427nM
Vorinostat	HDAC	1360 [26]	2066nM
JQ1	BRD4	8700 [27]	4869nM
LY2606368	CHK1	673 [28]	NA, <200nM [23]
PTC596	BMI1	4710 [29]	NA, <1200nM [24]
Bortezomib	proteasome	276 [30]	4.2nM
Dinacicilb	CDK1/2/5/9	4565 [31]	25.4nM
GDC-0941	PI3K	1520 [32]	1854nM

^+^https://www.cancerrxgene.org/compounds [25–32]

### Synergy studies

The 13 drug combinations that showed potent cell killing across multiple cell lines were selected for further synergy analysis. Fig 3D and 3E show the synergy plot of in the 5746 line for the GDC0941 and Vorinostat (HDAC inhibitor) combination therapy. Table 1 lists if the evaluated combinations are synergistic (Synergy Score δ>10), additive (Synergy Score -10<δ<10) or antagonistic (Synergy Score δ<-10). Compounds targeting BRD4, CHEK1 and BMI-1 worked synergistically with MEK inhibitors, compounds targeting HDACs, CHEK1 and BMI1 worked synergistically with PI3K inhibitors. The combination of MEK and PI3K inhibitors also synergistically inhibited cell growth.

## Discussion

High Grade Glioma in Neurofibromatosis type I is a devastating illness with no effective therapy. We derived and characterized glioma cell lines from 3 independent NPcis mice and evaluated potential therapeutic sensitivities. Importantly, these mouse lines express OPC lineage markers consistent with what is observed in many HGG. Crucially, our study includes the only NF1 patient derived HGG cell line available. This line has loss of NF1, ATRX, TP53 and CDKN2A, consistent with what is observed in other NF1-HGG [8, 10]. Moreover, markers expressed by the TM31 cell line are also consistent with an OPC lineage origin.

The sensitivity of our NF1-HGG lines to a range of anti-cancer compounds, inhibiting different targets, was evaluated alone and in combination with MEK and PI3K inhibitors. Importantly, the 3 mouse glioma lines were evaluated in a spheroid drug sensitivity screen. Non-serum grown glioma cell lines are considered more accurate models for glioma, moreover, compared to 2D, a 3D organoid like growth activates different signaling pathways and is considered more representative of *in vivo* models [13–15]. The screen focused on targets with drugs in clinical development and combination therapies with inhibitors that target MEK and PI3K were evaluated. Given the aberrant RAS pathway activation induced by loss of NF1, MEK or PI3K inhibitors will most likely form the backbone of any NF1 patient tumor treatment. GDC-0941 (Pictilisib) is part of a phase IIb clinical trial in patients with Glioblastoma/Gliosarcoma to evaluate the anti-neoplastic effect of the PD-1 monoclonal antibody pembrolizumab (MK-3475), in combination with PI3K inhibitors (NCT02430363). Similarly, Trametinib forms the backbone of several ongoing glioma clinical trials in combination with the BRAF inhibitor, dabrafenib (GSK-2118436), including pediatric patients with newly diagnosed HGG (NCT03919071) and NF1-LGG or LGG with RAS pathway aberrations (NCT03363217). Both Pictilisib and Trametinib hold a strong promise for translational potential. Excitingly, 6 drug targets (HDAC, BRD4, CHEK1, BMI-1, CDK1/2/5/9 and the proteasome) were identified that, alone or when inhibited in combination with MEK or PI3K inhibitors, caused potent cell death at low concentrations across most of the evaluated lines. The observed LD50 values were compared to the IC50 values of a large set of cancer cell lines (The Genomics of Drug Sensitivity in Cancer Project). Crucially, the observed LD50 values, i.e. the concentration that causes 50% cell death, were consistently at or below the 25^th^ percentile of the IC50 values, i.e. concentrations that cause 50% growth inhibition but no cell death, reported by The Genomics of Drug Sensitivity in Cancer Project in our mouse 5746 line. This is also true for most of the combinations evaluated in our human TM31 cell line (see Tables 1 and 2). Our results highlight the degree to which the NF1-HGG are sensitive to these drugs and suggests these could be viable targets.

Six drugs combined with MEK or PI3K and the combination of MEK and PI3K inhibitors worked synergistically in our mouse NF1-HGG stem cell line. None of the combinations worked synergistically in the human TM-31 line (12 additive and 1 antagonistic combination). Crucially however, baseline sensitivity to these compounds remains high in our human NF1 HGG line. Moreover, while synergistic effects of drug combinations point to a mechanistic interaction between both compounds, the fact that a combination therapy work additively should not exclude them from clinical trials or further investigation. It is unclear if the observed differences between our mouse and human line are due to differences in driver mutations (*ATRX* and *CDKN2a*), culture media (serum versus stem cell media), culture method (2D vs 3D assays) and/or species (human versus mouse) and will require the evaluation of other human NF1 HGG once they become available. As mentioned before, it is believed that 3D stem cell cultures represent more accurately the biology of tumors than 2D serum grown lines. Our screen provides valuable insight into potential drug targets for NF1 associated HGG. Follow-up *in vivo* pre-clinical studies will have to confirm if the identified drug targets and drug combinations are able to shrink tumors in an *in vivo* setting at well tolerated doses and enhance survival in preclinical models of NF1-HGG.

## Supporting information

S1 Fig Raw images of Figs 1–3. Raw and uncropped western blot and gel electrophoresis images of Figs 1–3.
(PDF)

S1 Table Evaluated drugs. Overview of the drugs used in this study including targets and catalogue numbers.
(XLSX)

S2 Table Log2 value of fold change in RFP intensity (spheroid lines 17, 5746 and 5653) or cell number (TM31). Drug sensitivity data expressed in log2 fold change in the different cell lines. A value <0 denotes that less cells are present compared to day 0.
(XLSX)

S3 Table 3D spheroid assay incucyte analysis settings. Incucyte analysis settings and parameters used to analyze our 3D spheroid assays.
(XLSX)

S4 Table 2D cell counting assay incucyte analysis settings. Incucyte analysis settings and parameters used to analyze our 3D spheroid assays.
(XLSX)
