# Supplementary material for: Identification of therapeutic sensitivities in a spheroid drug combination screen of Neurofibromatosis Type I associated High Grade Gliomas
Source: PLoS One. 2023 Feb 2;18(2):e0277305. doi: 10.1371/journal.pone.0277305 (PMC9894422; doi:10.1371/journal.pone.0277305)
Supplement: S1 Fig — Raw and uncropped western blot and gel electrophoresis images of Figs 1–3. (PDF) [file pone.0277305.s001.pdf]

Figure 1b: genotyping PCR

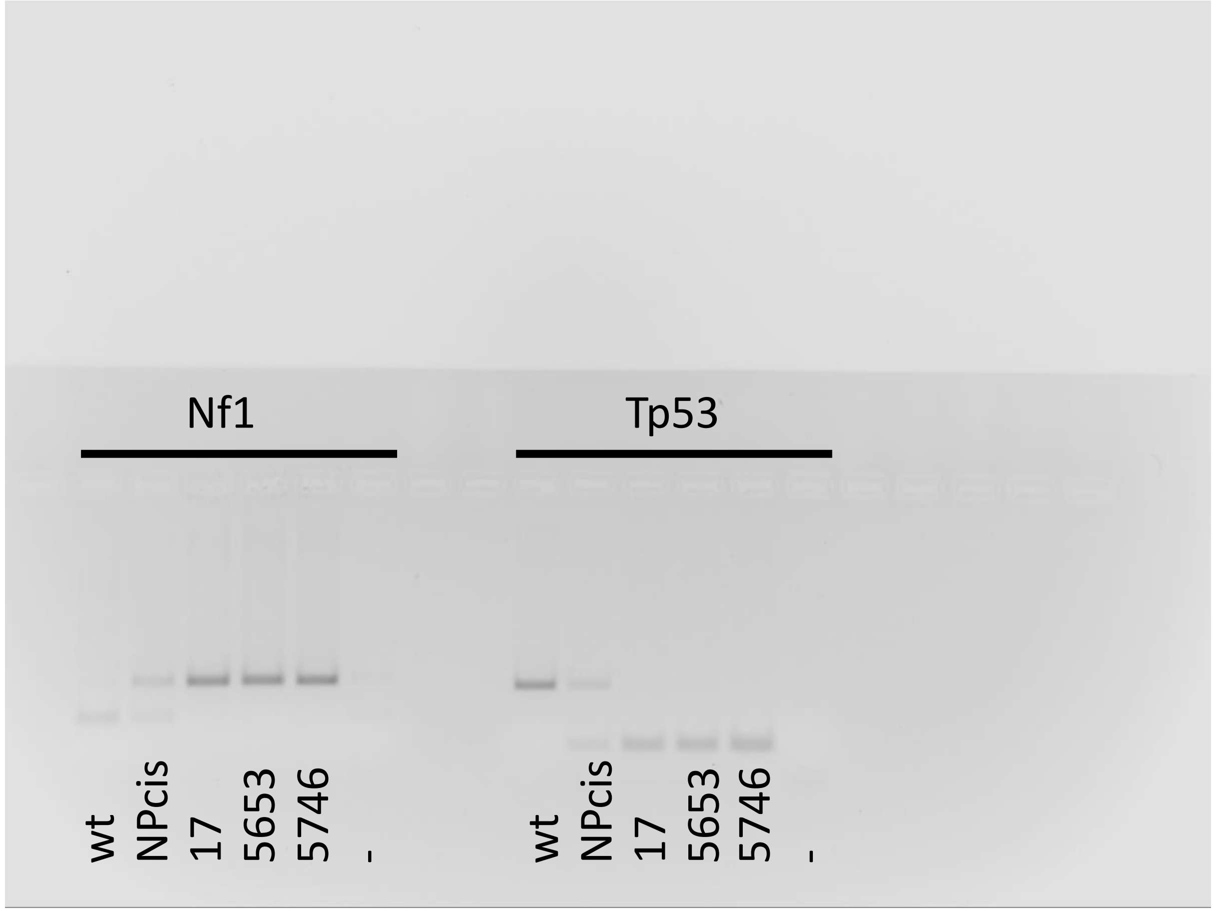

Figure 1c: OPC lineage western blot

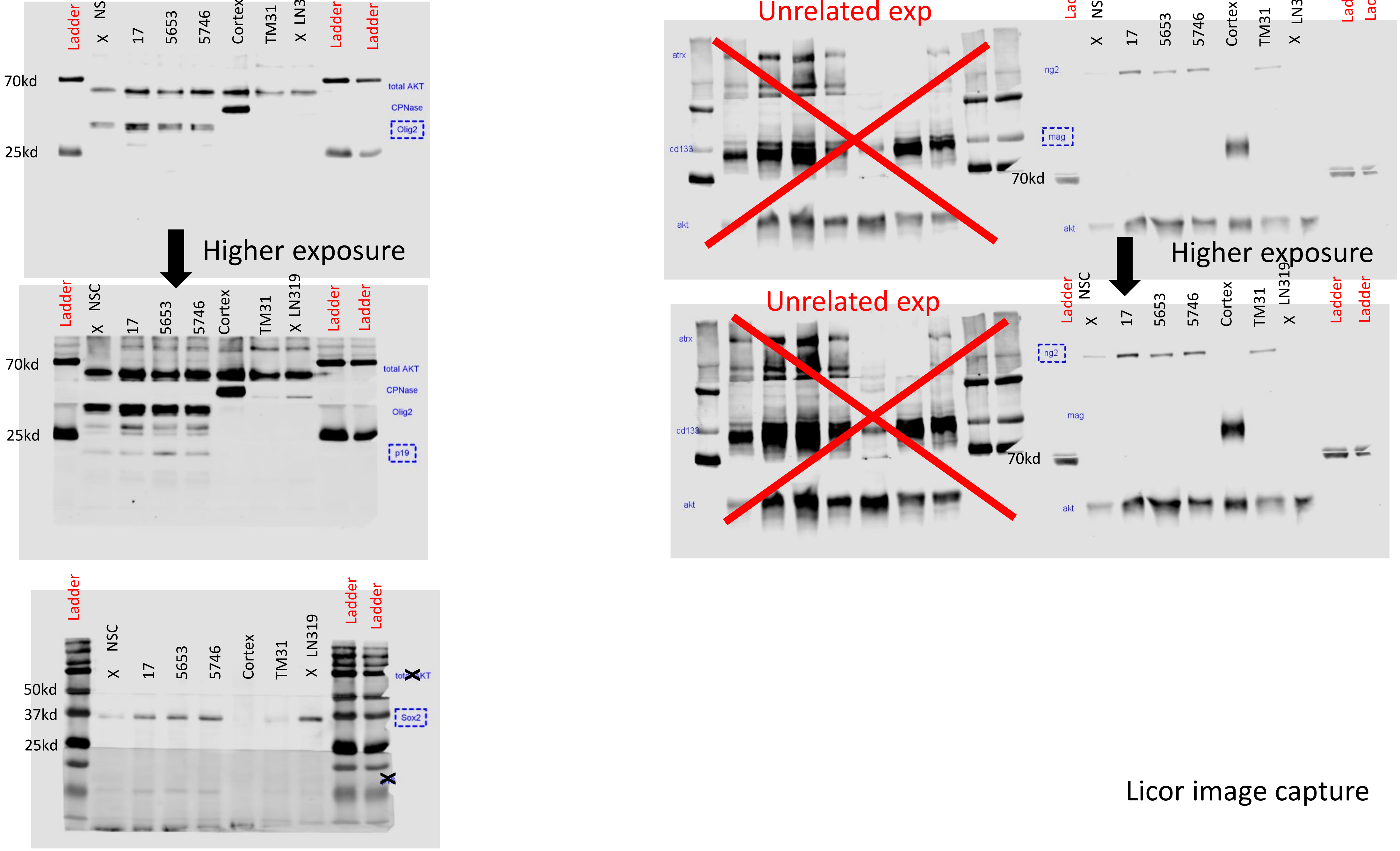

Figure 1f: TM31 western blot

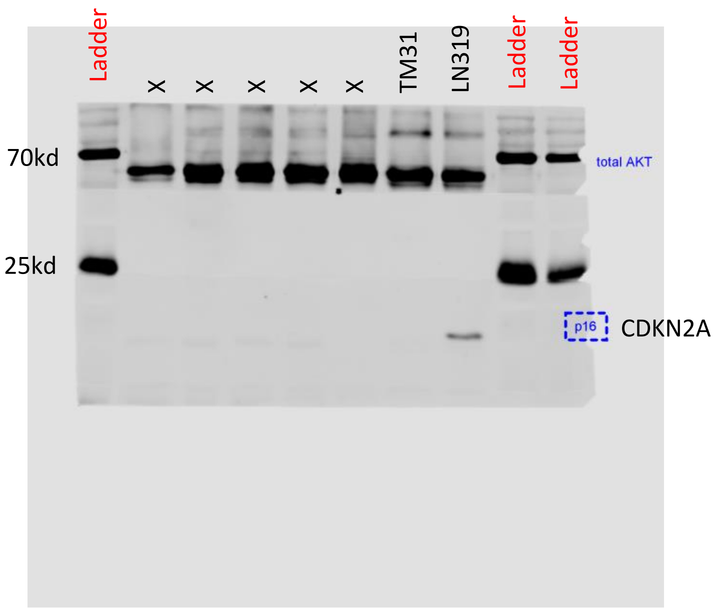

Lower exposure

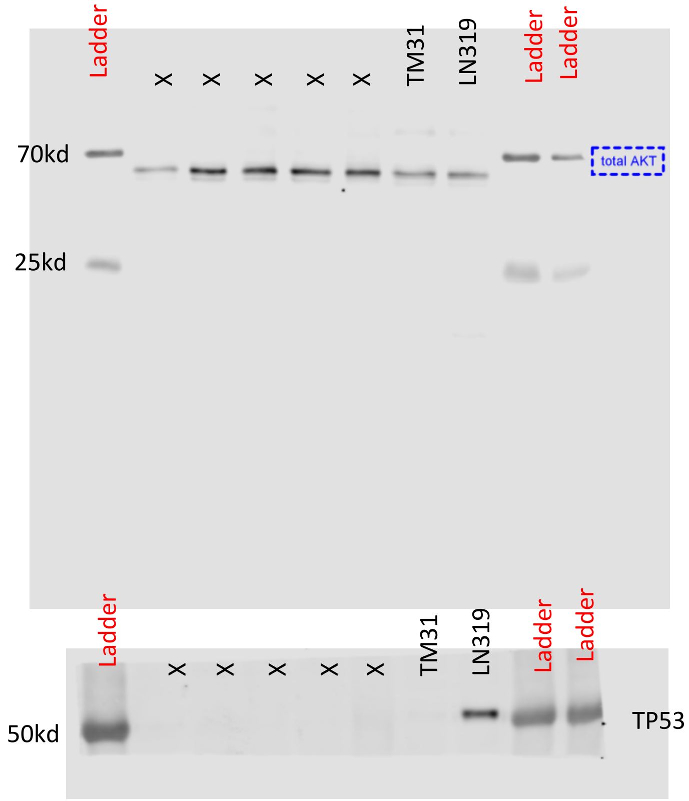

Licor image capture

Figure 2c: Western blot drug inhibition

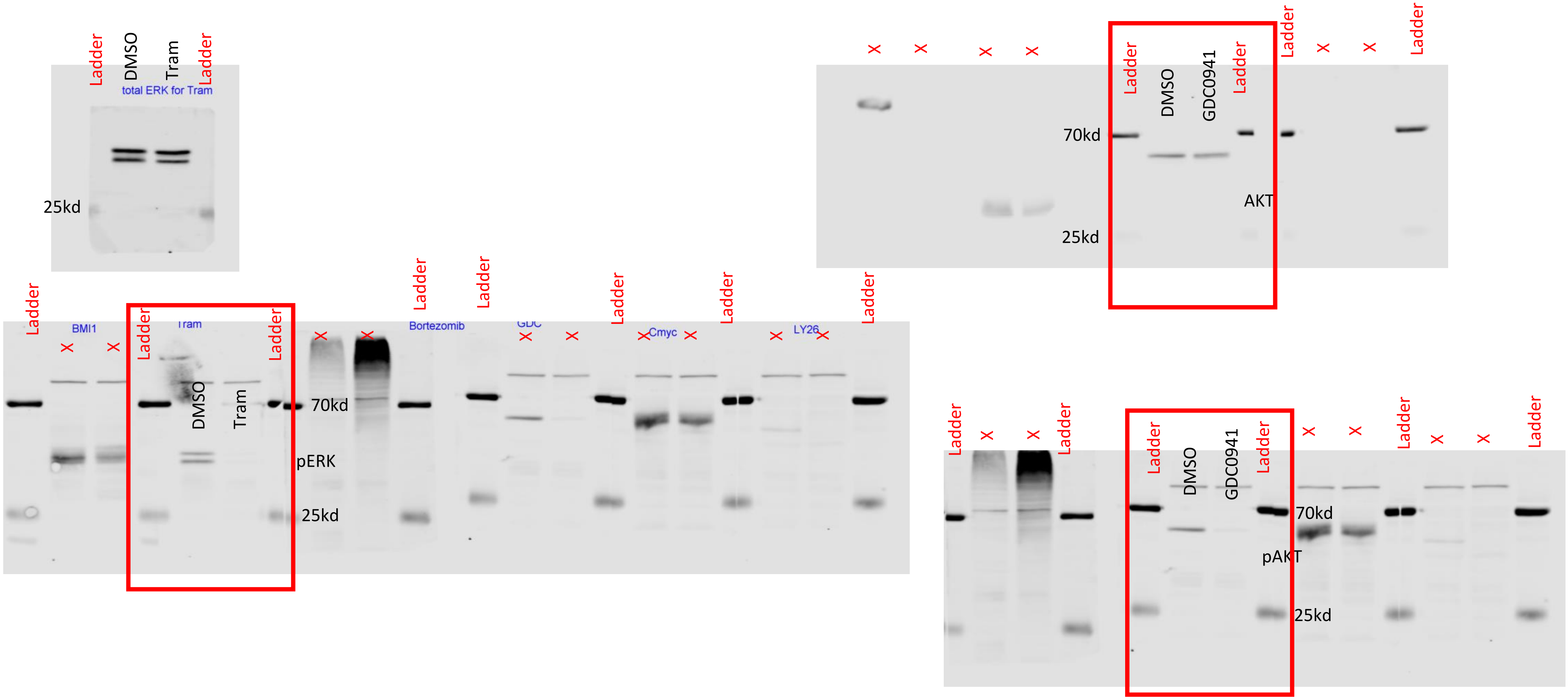

Licor image capture

Figure 3c: Western blot drug inhibition

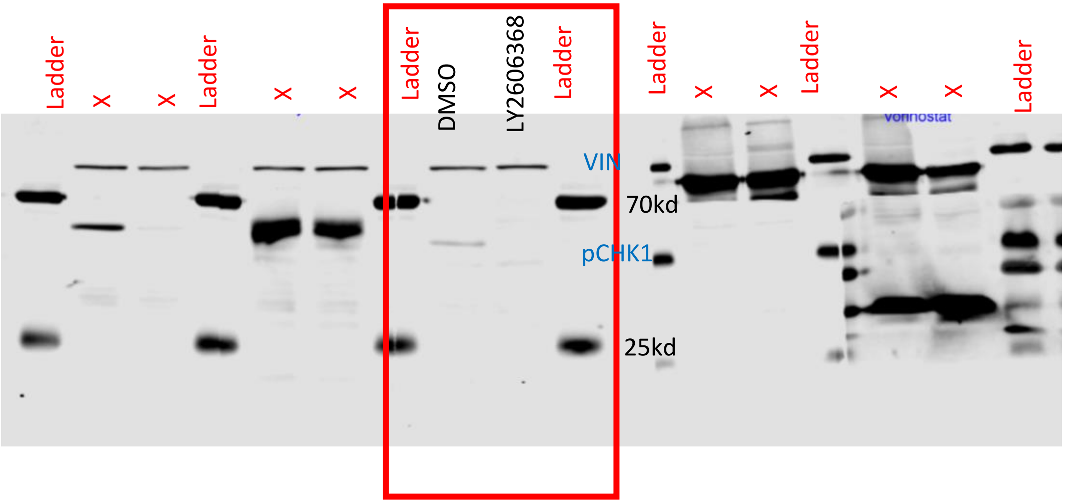

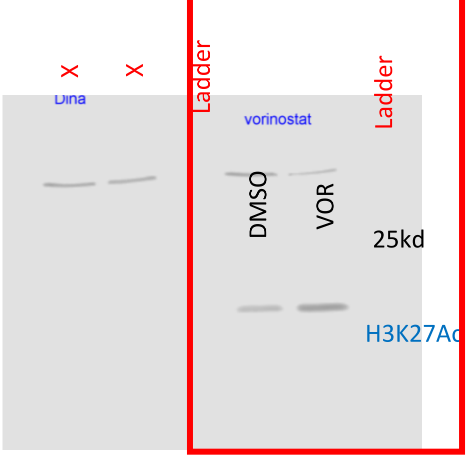

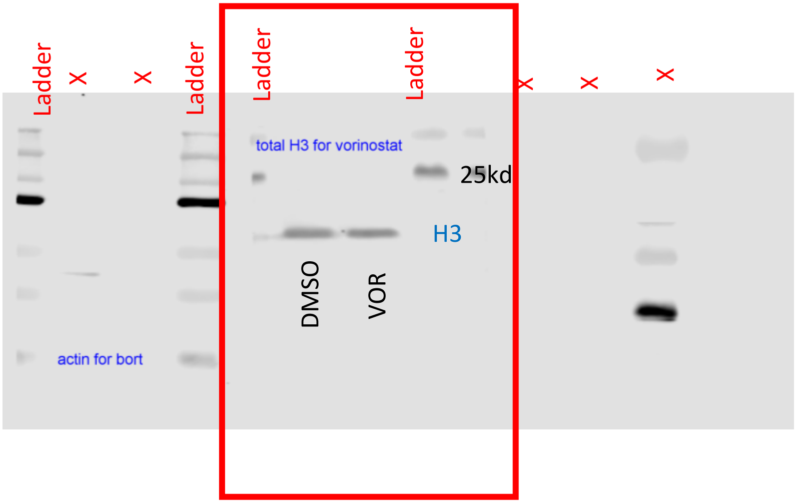

Licor image capture

Figure 3c: Western blot drug inhibition

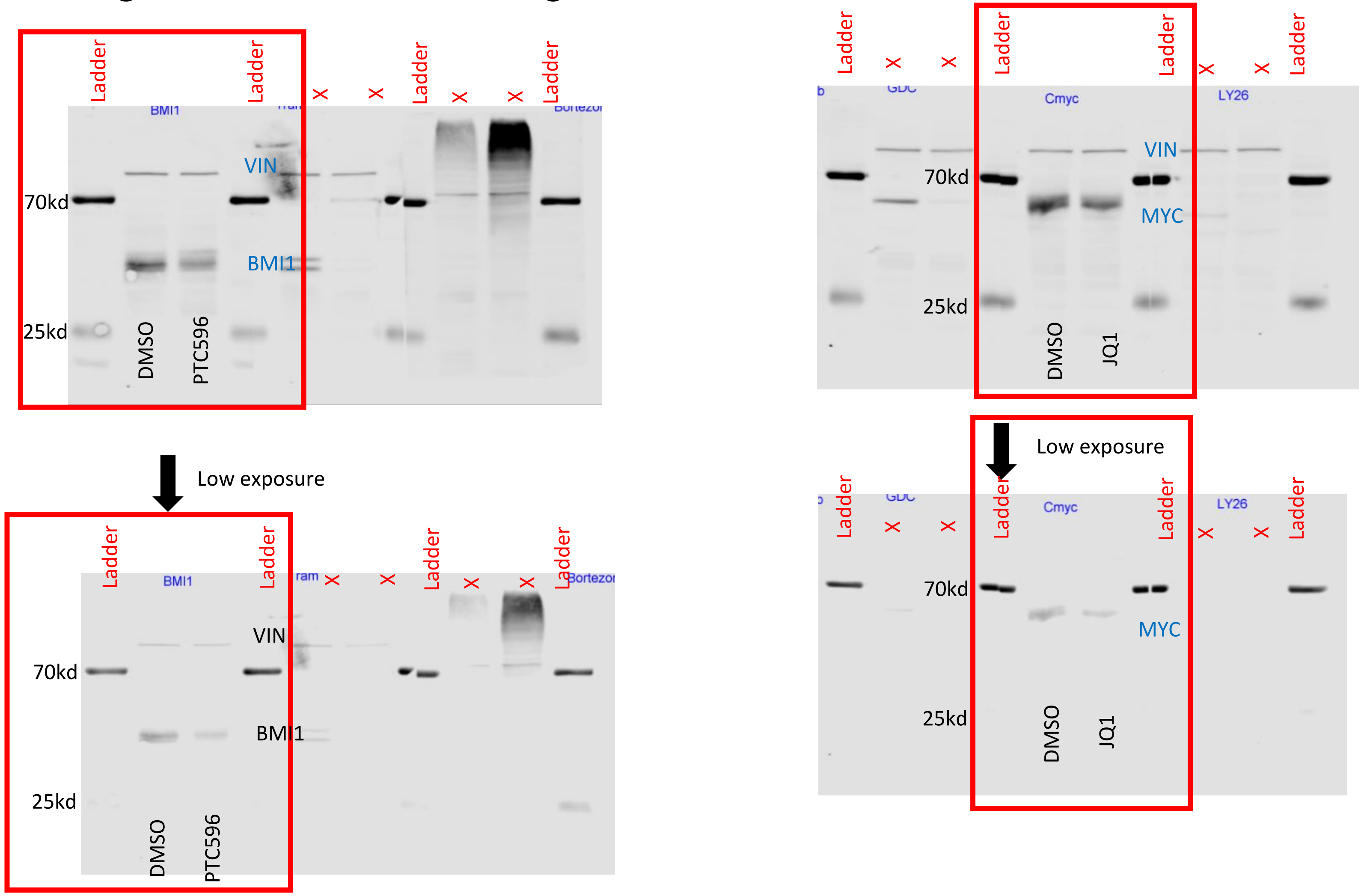

Licor image capture

Figure 3c: Western blot drug inhibition

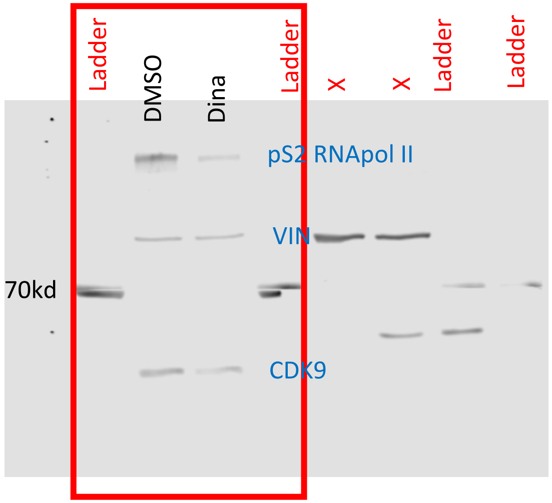

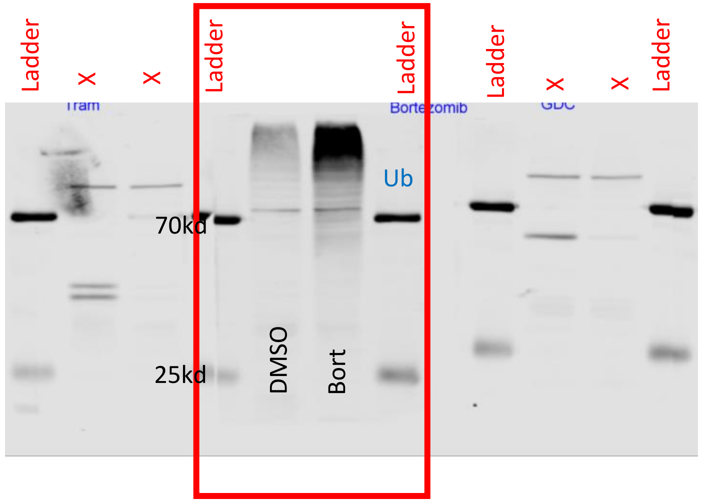

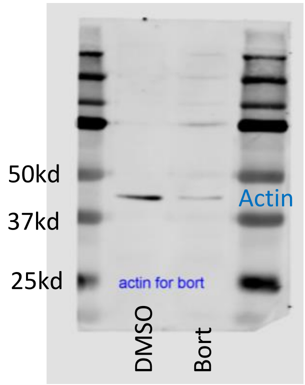

Licor image capture
